# Clinical features of chronic inflammatory demyelinating polyneuropathy: a single-center experience with 33 patients

**DOI:** 10.3389/fneur.2026.1743650

**Published:** 2026-02-26

**Authors:** Toshiya Nomura, Yohei Misumi, Mitsuharu Ueda

**Affiliations:** Department of Neurology, Graduate School of Medical Sciences, Kumamoto University, Kumamoto, Japan

**Keywords:** CIDP, CIDP variants, efgartigimod, IVIg, plasma exchange, SCIg, steroid, typical CIDP

## Abstract

**Background:**

Chronic inflammatory demyelinating polyneuropathy (CIDP) is an immune-mediated neuropathy with heterogeneous clinical presentations. Although several clinical subtypes, including typical and variants, have been described, evidence regarding their distribution and treatment outcomes remains limited, particularly for CIDP variants.

**Methods:**

We retrospectively analyzed 33 consecutive patients diagnosed with CIDP at Kumamoto University Hospital between January 2020 and March 2025. Clinical data included demographic characteristics, disease duration, subtype classification, electrophysiological and cerebrospinal fluid (CSF) findings, imaging findings, treatment details, and treatment responsiveness. Subtypes were classified according to the 2021 EAN/PNS diagnostic criteria. Treatment responsiveness was defined as objective improvement confirmed by at least two neurologists.

**Results:**

Among the 33 patients, 36.4% had typical CIDP and 63.6% had CIDP variants, including distal (39.4%), multifocal (15.2%), motor (3.0%), and sensory (6.0%) subtypes. Distal CIDP was the most frequent subtype. Patients with multifocal CIDP experienced the longest diagnostic delays (mean, 4.6 years) due to fewer demyelinating findings on electrophysiological studies. The MRC sum score was lowest in typical CIDP, suggesting greater disease severity. Intravenous immunoglobulin (IVIg) demonstrated high efficacy across all subtypes, including multifocal CIDP, in contrast to previous reports of lower responsiveness. During a mean follow-up period of 5.1 years, 78.8% of patients required maintenance therapy, most commonly IVIg and corticosteroids. No changes in clinical subtypes were observed during follow-up.

**Conclusion:**

In this single-center study, variants outnumbered typical CIDP, reflecting the case mix at a specialized tertiary referral center. Multifocal CIDP showed the longest diagnostic delays. IVIg demonstrated high efficacy across subtypes, including multifocal CIDP, contrasting with previous reports. These findings highlight the importance of improving diagnostic accuracy and establishing individualized long-term treatment strategies for CIDP.

## Introduction

Chronic inflammatory demyelinating polyneuropathy (CIDP) is an immune-mediated peripheral neuropathy characterized by progressive or relapsing–remitting weakness and sensory dysfunction resulting from demyelination of peripheral nerves. While typical CIDP is the most widely recognized phenotype, several variants have also been described, including distal, multifocal, focal, motor, and sensory CIDP. These clinical subtypes exhibit heterogeneity in symptom distribution, electrophysiological findings, cerebrospinal fluid profiles, and responsiveness to immunotherapies (1–3).

Most previous studies have reported that typical CIDP accounts for approximately 50–60% of cases, whereas CIDP variants comprise the remaining 40%–50% (2–7). However, the frequency and clinical profiles of these variants vary among studies, possibly owing to differences in diagnostic criteria, disease stage, or patient selection. Importantly, patients with CIDP variants often experience longer diagnostic delays than those with typical CIDP (8–10). Such diagnostic delays may lead to postponed treatment initiation and poorer clinical outcomes.

Treatment responsiveness also differs among subtypes. Intravenous immunoglobulin (IVIg) and corticosteroids are established first-line therapies for CIDP, but previous reports have suggested that IVIg is less effective in multifocal CIDP than in typical CIDP (2, 3, 11). The reasons for these differences remain unclear but may involve distinct underlying pathophysiological mechanisms—humoral immune processes in typical CIDP versus predominantly cellular mechanisms in multifocal CIDP (12, 13). Early diagnosis and prompt initiation of treatment with IVIg, corticosteroids, or plasma exchange are essential to prevent long-term disability in CIDP. Recently, novel molecular-targeted therapies, such as anti-FcRn monoclonal antibodies, have demonstrated significant efficacy in patients with CIDP (14). Nevertheless, clinical heterogeneity continues to hinder early diagnosis and timely initiation of appropriate treatment.

Therefore, this retrospective, single-center study aimed to investigate the distribution of CIDP subtypes, characterize their clinical features, and evaluate treatment responsiveness in patients diagnosed at Kumamoto University Hospital.

## Methods

We retrospectively analyzed the clinical data of 33 consecutive patients diagnosed with CIDP at our hospital between January 1, 2020, and March 31, 2025. The diagnosis of CIDP was made by neurologists according to the 2021 EAN/PNS diagnostic criteria (1). The clinical data included age at onset, sex, duration from onset to diagnosis, clinical subtype, MRC scale scores, cerebrospinal fluid findings, electrophysiological findings, imaging findings, treatment details, and treatment responsiveness. IVIg was administered as induction therapy at a total dose of 2.0 g/kg over 5 days, followed by maintenance therapy at 1.0 g/kg every 3–6 weeks, depending on clinical response. Treatment responsiveness was defined as objective improvement in grip strength, manual muscle testing (MMT), sensation, or gait, as confirmed by at least two neurologists. Descriptive analysis was performed for baseline patient characteristics and clinical outcomes, with number and proportion (%) for categorical data and mean with standard deviation (SD) for continuous data. All statistical analyses were performed using Microsoft Excel 2021 (Microsoft Corporation, Redmond, WA, USA), the “meta” package in R Statistical Software (version 4.1.3), and RStudio.

## Results

**Figure 1:**
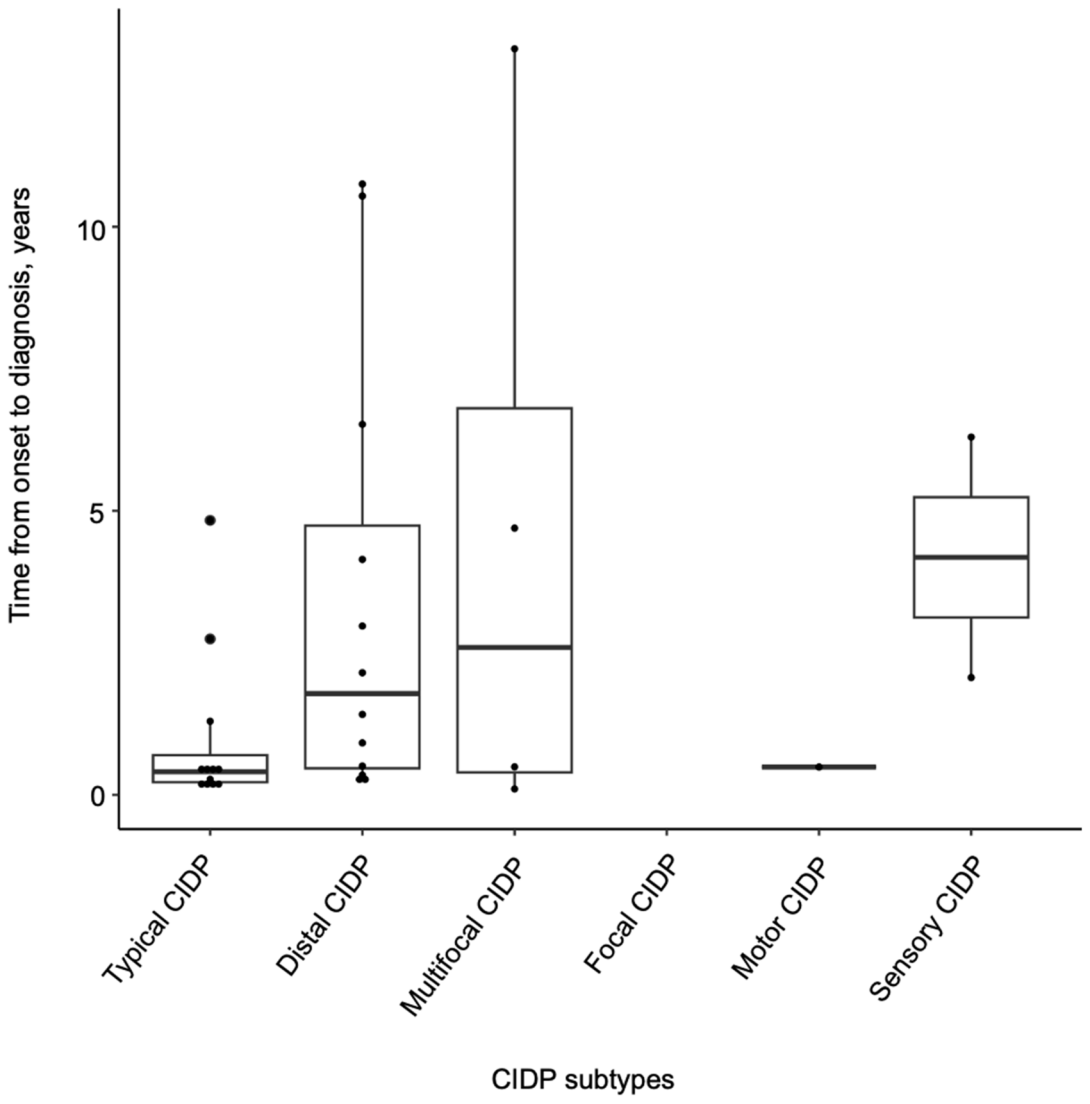
Impact of subtypes on diagnosis timing. This figure analyzes the diagnostic intervals in 33 patients with CIDP, based on the subtypes. Categories include typical, distal, multifocal, focal, motor, and sensory CIDP, showing the influence of subtypes on diagnostic delays.

Table 1 depicts the demographic and clinical characteristics of patients with CIDP, classified by CIDP subtypes. Among the 33 patients, the distribution of the subtypes was 36.4% for typical CIDP (*n* = 12), 39.4% for distal CIDP (*n* = 13), 15.2% for multifocal CIDP (*n* = 5), 3.0% for motor CIDP (*n* = 1), and 6.0% for sensory CIDP (*n* = 2). In this study, distal CIDP was the most common subtype. The gender distribution was nearly equal (17 males and 16 females), and the mean age at onset was 47 ± 17 years. The MRC sum score was lowest in patients with typical CIDP, suggesting greater disease severity in this subtype. Patients with distal CIDP, the most frequent subtype in this cohort, showed a lower age at onset and a male predominance compared with other CIDP subtypes. The MRC sum score was relatively preserved in patients with distal CIDP; however, clinically evident distal muscle weakness involving the fingers and toes, which is not adequately captured by the MRC sum score, was observed. The mean time from onset to diagnosis in patients with CIDP was analyzed according to the subtypes. The shortest time to diagnosis was observed in patients with typical CIDP (1.0 ± 1.4 years), whereas patients with multifocal CIDP experienced the longest diagnostic delay (4.6 ± 6.1 years) (Table 1; Figure 1). Patients with multifocal CIDP were initially misdiagnosed as having drug-induced neuropathy, idiopathic neuropathy, cervical spondylosis, or tarsal tunnel syndrome.

**Table 1:** Demographic and clinical characteristics.

	All patients	Typical CIDP	Distal CIDP	Multifocal CIDP	Focal CIDP	Motor CIDP	Sensory CIDP
Patients, *n* (%)	33 (100%)	12 (36.4%)	13 (39.4%)	5 (15.2%)	0 (0%)	1 (3.0%)	2 (6.0%)
Age, mean ± SD (years)	57 ± 17	60 ± 18	53 ± 18	60 ± 11	NA	64	64 ± 13
Sex, *n* (%)							
Male	17 (51.5%)	5 (41.7%)	9 (69.2%)	1 (20%)	NA	1 (100%)	1 (50%)
Female	16 (48.5%)	7 (58.3%)	4 (30.8%)	4 (80%)	NA	0 (0%)	1 (50%)
Onset age, mean ± SD (years)	47 ± 17	52 ± 18	42 ± 15	45 ± 20	NA	61	55 ± 18
Time from onset to diagnosis, mean ± SD (years)	2.6 ± 3.5	1.0 ± 1.4	3.4 ± 3.9	4.6 ± 6.1	NA	0.5	4.2 ± 3.0
MRC sum score, mean ± SD	51.7 ± 9.6	43.8 ± 10.8	56.5 ± 3.3	54.8 ± 4.6	NA	60	60 ± 0
MRC score (upper limbs), mean ± SD	26.1 ± 4.6	21.9 ± 4.1	29.3 ± 2.1	26.5 ± 3.5	NA	30	30 ± 0
MRC score (lower limbs), mean ± SD	25.6 ± 5.8	21.9 ± 7.7	27.3 ± 2.6	28.3 ± 1.3	NA	30	30 ± 0

This table presents the demographic and clinical characteristics of CIDP, classified by the subtype.

**Figure 2:**
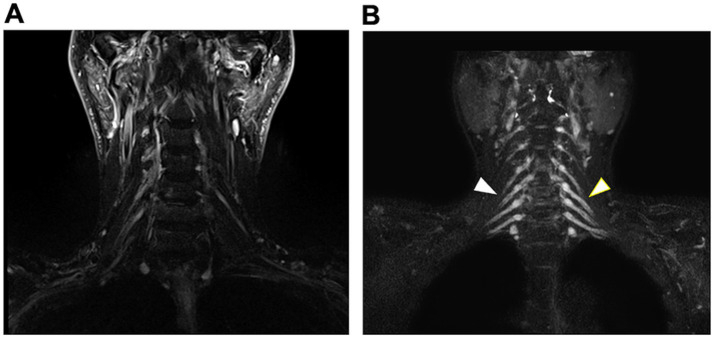
Representative MR neurography findings in a patient with CIDP. **(A)** Coronal STIR image showing no hypertrophy of the cervical nerve roots in a patient with typical CIDP. **(B)** Coronal STIR image demonstrating marked enlargement of the cervical nerve roots (arrowheads) in a patient with typical CIDP.

The laboratory findings are summarized in Table 2. Electrophysiological findings varied across CIDP subtypes. The lowest motor nerve conduction velocity, along with prolonged distal latency and F-wave latency, was observed in patients with typical CIDP. In addition, the compound muscle action potential amplitudes in this subtype were the lowest among all subtypes, suggesting greater disease severity. In contrast, distal latency and F-wave latency were relatively preserved in patients with multifocal CIDP. Reduced SNAP amplitudes in the lower limbs were observed in patients with distal CIDP compared with other CIDP subtypes, with a paucity of typical demyelinating features. CSF protein levels were higher in typical CIDP than in other subtypes. Figure 2 shows representative MR neurography images demonstrating abnormal findings, particularly nerve root enlargement. The diagnostic sensitivity of MR neurography was 74.2%, varying among subtypes (Table 2; Figure 2).

**Table 2:** The laboratory findings.

	All patients	Typical CIDP	Distal CIDP	Multifocal CIDP	Focal CIDP	Sensory CIDP	Sensory CIDP
MCS Median nerve, mean ± SD							
Distal latency, ms	8.0 ± 5.6	11.1 ± 7.9	6.6 ± 3.0	4.7 ± 0.8	NA	7.4	6.7 ± 1.6
CV, m/s	36.8 ± 12.3	27.7 ± 15.9	39.5 ± 13.5	33.8 ± 16.2	NA	30.3	48.0 ± 0.9
CMAP, mV	5.1 ± 3.7	2.6 ± 2.2	6.5 ± 3.1	7.6 ± 6.5	NA	4.3	5.1 ± 3.4
F-wave latency, ms	44.4 ± 15.2	53.1 ± 17.7	43.9 ± 13.2	29.0 ± 6.1	NA	40.8	37.0^a^
MCS Tibial nerve, mean ± SD							
Distal latency, ms	7.8 ± 5.7	10.8 ± 6.5	7.3 ± 6.0	5.0 ± 1.3	NA	4.7	4.0 ± 0.4
CV, m/s	37.7 ± 9.7	10.8 ± 6.5	37.1 ± 9.9	41.9 ± 5.3	NA	34.0	39.4 ± 0.2
CMAP, mV	5.9 ± 5.3	3.0 ± 3.2	6.1 ± 7.2	6.9 ± 4.6	NA	4.3	6.2 ± 4.9
F-wave latency, ms	65.8 ± 21.1	84.6 ± 26.9	62.3 ± 20.0	53.8 ± 11.5	NA	96.4	57.2 ± 2.7
SCS Median nerve, mean ± SD							
CV, m/s	49.9 ± 17.3	23.4 ± 27.2	47.4 ± 16.5	61.4 ± 23.1	NA	ND	51.5^a^
SNAP, μV	3.9 ± 3.0	2.0 ± 2.4	4.9 ± 3.8	2.5 ± 2.1	NA	ND	0.4^a^
SCS Sural nerve, mean ± SD							
CV, m/s	46.1 ± 9.6	27.0 ± 24.1	45.5 ± 14.3	48.6 ± 4.6	NA	44.3	41.2^a^
SNAP, μV	11.4 ± 8.7	9.3 ± 9.8	6.9 ± 10.0	9.9 ± 5.6	NA	4.5	11.4^a^
CSF protein, mean ± SD (mg/dL)	105.3 ± 67.4	140.2 ± 66.9	96.6 ± 65.8	64.9 ± 51.5	NA	63.8	45.4 ± 21.4
MRI abnormalities/tested, *n* (%)	23/31 (74.2%)	8/12 (66.7%)	10/11(90.9%)	4/5 (80%)	NA	0/1 (0%)	1/2 (50%)

a Standard deviation could not be calculated due to undetectable values.

MCS, motor conduction study; CV, conduction velocity; CMAP, compound muscle action potential; SCS, sensory conduction study; SNAP, sensory nerve action potential; CSF, cerebrospinal fluid; NA, not applicable; ND, not determined.

Normal reference values for NCS were defined as follows: motor conduction velocity (wrist-elbow) ≥ 50 m/s (median nerve) and motor conduction velocity (ankle-popliteal) ≥ 40 m/s (tibial nerve); distal motor latency ≤ 4.6 ms (median nerve) and ≤ 5.7 ms (tibial nerve); CMAP amplitude ≥ 3.0 mV (median nerve) and ≥ 4.3 mV (tibial nerve); F wave latency ≤ 28.2 ms (median nerve) and ≤ 51.7 ms (tibial nerve); SNAP amplitude ≥ 7 μV (median nerve) and ≥ 7.4 μV (sural nerve).

Normal reference range for CSF protein was 8–43 mg/dL.

This table presents information on the laboratory findings of CIDP.

Table 3 shows the details of induction treatments and treatment responsiveness.

**Table 3:** Induction treatments and treatment responsiveness.

	All patients	Typical CIDP	Distal CIDP	Multifocal CIDP	Focal CIDP	Motor CIDP	Sensory CIDP
IVIg							
Treated, *n* (%)	32/33 (97.0%)	12/12 (100%)	13/13 (100%)	5/5 (100%)	NA	1/1 (100%)	1/2 (50%)
Response, *n* (%)	31/32 (96.9%)	11/12 (91.7%)	13/13 (100%)	5/5 (100%)	NA	1/1 (100%)	1/1 (100%)
Steroid							
Treated, *n* (%)	11/33 (33.3%)	6/12 (50%)	3/13 (23.1%)	1/5 (20%)	NA	0/1 (0%)	1/2 (50%)
Response, *n* (%)	10/11 (90.9%)	5/6 (83.3%)	3/3 (100%)	1/1 (100%)	NA	NA	1/1 (100%)
PE							
Treated, *n* (%)	3/33 (9.1%)	3/12 (25%)	0/13 (0%)	0/5 (0%)	NA	0/1 (0%)	0/2 (0%)
Response, *n* (%)	3/3 (100%)	3/3 (100%)	NA	NA	NA	NA	NA

IVIg, intravenous immunoglobulin; PE, plasma exchange; NA, not applicable.

This table presents induction treatments and treatment responsiveness, classified by CIDP subtype.

As induction therapy, IVIg was most frequently used (32 cases, 97.0%), followed by corticosteroids (11 cases, 33.3%) and plasma exchange (3 cases, 9.1%). Overall, both IVIg and corticosteroids demonstrated high efficacy, with response rates of 96.9% and 90.9%, respectively. However, a subset of patients with typical CIDP (3 cases, 25%) showed treatment resistance and required plasma exchange. In contrast, patients with CIDP variants, such as multifocal CIDP and distal CIDP, generally showed a favorable response to both IVIg and corticosteroids (Table 3).

During a mean follow-up period of 5.1 years, 7 patients (21.2%) did not require maintenance therapy, whereas 26 patients (78.8%) continued maintenance treatment. Intravenous immunoglobulin (IVIg) and corticosteroids were the most commonly used maintenance therapies (13 patients, 52%), followed by subcutaneous immunoglobulin (SCIg) (2 patients, 6.1%) and efgartigimod (1 patient, 3%) (Table 4). During this follow-up period, no patients exhibited a change in their clinical subtype at the final evaluation.

**Table 4:** Maintenance treatments.

	All patients	Typical CIDP	Distal CIDP	Multifocal CIDP	Focal CIDP	Motor CIDP	Sensory CIDP
Steroid							
Treated, *n* (%)	13/33 (39.4%)	7/12 (58.3%)	3/13 (23.1%)	1/5 (20%)	NA	0/1 (0%)	2/2 (100%)
IVIg							
Treated, *n* (%)	13/33 (39.4%)	5/12 (41.7%)	6/13 (46.2%)	2/5 (40%)	NA	2/5 (40%)	0/2 (0%)
SCIg							
Treated, *n* (%)	2/33 (6.1%)	1/12 (8.3%)	1/13 (7.7%)	0/5 (0%)	NA	0/1 (0%)	0/2 (0%)
Efgartigimod							
Treated, *n* (%)	1/33 (3%)	0/12 (0%)	0/13 (0%)	1/5 (20%)	NA	0/1 (0%)	0/2 (0%)
No maintenance treatment							
*n* (%)	7/33 (21.2%)	1/12 (8.3%)	3/13 (23.1%)	2/5 (40%)	NA	1/1 (100%)	0/2 (0%)

IVIg, intravenous immunoglobulin; SCIg, subcutaneous immunoglobulin; NA, not applicable.

This table shows maintenance treatments, classified by CIDP subtype.

## Discussion

This retrospective, single-center study revealed that patients with CIDP variants were more common than those with typical CIDP. Clinical characteristics differed among CIDP subtypes. Patients with multifocal CIDP experienced diagnostic delays due to the paucity of demyelinating findings on electrophysiological studies. Additionally, IVIg demonstrated high efficacy even in patients with multifocal CIDP, in contrast to previous studies that have reported lower responsiveness in this subtype (2, 3, 11).

In our cohort, CIDP variants were more frequent than typical CIDP. Our study showed that typical CIDP accounted for 36.4% of patients, whereas CIDP variants—including distal, multifocal, motor, and sensory CIDP—accounted for 63.6%. Most previous studies have reported that typical CIDP accounts for approximately 50%–60% of patients (2–7). Our findings differed from those of previous reports. In addition, distal CIDP was the most common subtype. The reason is not fully understood, but there are several possible explanations for this. First, our single center has an Amyloidosis Center, which assists clinicians in diagnosing and treating patients with ATTRv amyloidosis, a condition that should be differentiated from CIDP, particularly distal CIDP (15). It is possible that some patients for whom ATTRv amyloidosis was considered a differential diagnosis were ultimately diagnosed with CIDP. Second, increased clinical awareness of CIDP variants and the application of the updated EFNS/PNS diagnostic criteria may have facilitated the recognition of CIDP variants that might previously have been underdiagnosed. However, despite this increased awareness, CIDP variants remain diagnostically challenging because they often lack typical demyelinating features on electrophysiological studies. Consequently, patients with CIDP variants are more likely to experience diagnostic delays or apparent treatment resistance and are therefore referred to specialized centers, such as our hospital. Patients with CIDP variants, particularly those with multifocal CIDP, experienced a longer diagnostic delay than those with typical CIDP. The diagnostic delays observed in patients with CIDP variants were consistent with previous reports (8–10). The differences in diagnostic delay may be related to variations in electrophysiological findings based on the underlying pathophysiological mechanisms. In our study, as well as in previous reports, slowing of motor nerve conduction velocity, indicative of middle-segment involvement, was observed in patients with typical CIDP. Moreover, prolonged distal latency and F-wave latency, reflecting involvement of both distal and proximal segments where the blood–nerve barrier is deficient, were characteristic findings in typical CIDP (12, 13). In contrast, distal latency and F-wave latency tended to be relatively preserved in patients with multifocal CIDP, likely because the affected nerve trunks are located in regions where the blood–nerve barrier remains intact (13). Patients with multifocal CIDP are frequently misdiagnosed or underdiagnosed due to the paucity of demyelinating findings on electrophysiological studies, highlighting the importance for clinical awareness to improve diagnostic accuracy.

In our study, IVIg and corticosteroids demonstrated high efficacy, with response rates of 96.9% and 90.9%, respectively. Despite the high overall efficacy, treatment-resistant cases were observed, particularly among patients with typical CIDP. Furthermore, plasma exchange was more frequently required in severe cases of typical CIDP, suggesting that more aggressive therapeutic strategies may be necessary for this subgroup. Previous studies have reported that treatment responsiveness varies among CIDP subtypes, with IVIg generally considered more effective in typical CIDP than in multifocal CIDP (2, 3, 11). These differences may be related to distinct underlying pathophysiological mechanisms; antibody-mediated humoral immunity is thought to play a major role in typical CIDP, whereas cellular immunity may be more relevant in multifocal CIDP (12, 13). However, our findings differ from these previous reports, as IVIg was highly effective in patients with multifocal CIDP, achieving a 100% response rate in our cohort.

Our study demonstrated that, over a mean follow-up period of 5.1 years, the majority of patients (78.8%) required maintenance therapy. IVIg and corticosteroids were the predominant maintenance options, followed by SCIg and efgartigimod, highlighting the evolving landscape of long-term management strategies (16, 17). In an 8-year observational study, the proportion of CIDP variants decreased, whereas the proportion of typical CIDP increased (3). In the present study, no patients exhibited a change in their subtype during follow-up, which may be attributed to the prevention of disease progression by maintenance therapy or to the relatively short observation period. These findings emphasize the importance of personalized and sustained treatment strategies, as well as careful longitudinal monitoring.

This study has several limitations. First, the sample size was relatively small, particularly for motor and sensory CIDP. Second, as this was a retrospective analysis, potential selection bias cannot be excluded. Third, the follow-up duration in our study was relatively short, and the distribution of CIDP subtypes may differ over a longer follow-up period.

## Conclusion

In this single-center study of 33 CIDP patients, variants outnumbered typical CIDP, reflecting the case mix at a specialized tertiary referral center. Patients with multifocal CIDP experienced the longest diagnostic delays due to fewer demyelinating findings. IVIg demonstrated high efficacy across subtypes, including multifocal CIDP, in contrast to previous reports. These findings highlight the importance of improving diagnostic accuracy and establishing individualized long-term treatment strategies.

## Data Availability

The original contributions presented in the study are included in the article/supplementary material, further inquiries can be directed to the corresponding author.
